# Chemoradiotherapy with paclitaxel liposome plus cisplatin for locally advanced esophageal squamous cell carcinoma: A retrospective analysis

**DOI:** 10.1002/cam4.5416

**Published:** 2022-11-22

**Authors:** Qiong Yi, Canyu Liu, Yingshan Cui, Yanguang Yang, Yaqi Li, Xingwen Fan, Kailiang Wu

**Affiliations:** ^1^ Department of Radiation Oncology Fudan University Shanghai Cancer Center Shanghai China; ^2^ Department of Radiation Oncology Nantong Tumor Hospital, Affiliated Tumor Hospital of Nantong University Nantong China; ^3^ Department of Radiation Oncology Suzhou Dushu Lake Hospital, Dushu Lake Hospital Affiliated to Soochow University, Medical Center of Soochow University Suzhou China; ^4^ Department of Radiation Oncology Jinhua Hospital, Zhejiang University School of Medicine, Jinhua Municipal Central Hospital Zhejiang China; ^5^ Department of Oncology Shanghai Medical College, Fudan University Shanghai China; ^6^ Shanghai Clinical Research Center for Radiation Oncology Shanghai China; ^7^ Shanghai Key Laboratory of Radiation Oncology Shanghai China

**Keywords:** chemoradiotherapy, esophageal squamous cell carcinoma, paclitaxel liposome

## Abstract

**Purpose:**

This single‐center retrospective clinical study aimed to evaluate the efficacy and feasibility of chemoradiotherapy with paclitaxel liposome plus cisplatin for locally advanced esophageal squamous cell carcinoma (ESCC).

**Methods:**

Patients with locally advanced ESCC treated with paclitaxel‐liposome‐based chemoradiotherapy between 2016 and 2019 were retrospectively analyzed. Overall survival (OS) and progression‐free survival (PFS) were evaluated using Kaplan–Meier analysis.

**Results:**

Thirty‐nine patients with locally advanced ESCC were included in this study. The median follow‐up time was 31.5 months. The median OS time was 38.3 (95% confidence interval [CI]: 32.1–45.1) months, and the 1‐, 2‐, and 3‐year OS rates were 84.6%, 64.1%, and 56.2%, respectively. The median PFS time was 32.1 (95% CI: 25.4–39.0) months, and the 1‐, 2‐, and 3‐year PFS rates were 71.8%, 43.6%, and 43.6%, respectively. The most common Grade IV toxicity was neutropenia (30.8%) followed by lymphopenia (20.5%). There were no cases of Grade III/IV radiation pneumonia, and four patients (10.3%) had Grade III/IV esophagitis.

**Conclusion:**

Chemoradiotherapy using paclitaxel liposome and cisplatin is a well‐tolerated and effective treatment regimen for locally advanced ESCC.

## Graphical abstract

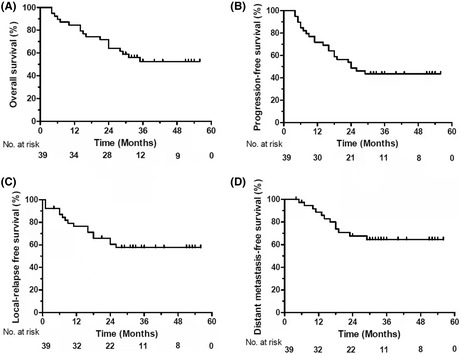

## 1 INTRODUCTION

Esophageal cancer is the seventh most common cancer and the sixth leading cause of cancer‐related mortality worldwide.^1^, ^2^, ^3^, ^4^ Squamous cell carcinoma is the main pathological type of esophageal cancer, accounting for 90% of all cases, particularly in China.^5^ For patients with locally advanced esophageal squamous cell carcinoma (ESCC) who are unsuitable for surgery, concurrent radiotherapy and chemotherapy are the standard treatments, with a 5‐year survival rate of approximately 30%–40%.^6^, ^7^, ^8^, ^9^, ^10^ Cisplatin plus fluorouracil (PF) has become the conventional chemotherapy regimen since the RTOG85‐01 clinical trial.^6^ However, toxicity limits the completion of concurrent chemotherapy,^11^ and the disease failure rates remain high, necessitating more effective regimens. Several protocols have been explored, including fluorouracil plus leucovorin and oxaliplatin (FOLFOX), paclitaxel and fluorouracil (TF), and paclitaxel and carboplatin (TC) schemes, which did not show significant survival benefits.^7^, ^10^, ^12^ Currently, paclitaxel‐based chemotherapy is a concurrent regimen commonly used in clinics. Especially for the paclitaxel and cisplatin (TP) regimen, has a lower incidence of radiation esophagitis and better tolerance and convenience compared with the classic PF regimen.^8^, ^10^, ^12^

Paclitaxel is a promising esophageal cancer chemotherapy drug, with an efficacy of approximately 32% as monotherapy in patients with locally advanced and metastatic esophageal cancer.^13^ Paclitaxel inhibits tubulin dissociation, thereby inhibiting mitosis, which blocks cell proliferation during the G2/M phase of the cell cycle and promotes the multipolar division of tumor cells.^14^ Paclitaxel is considered to be a good radiosensitizer; however, cremophor EL, a paclitaxel solvent, makes it prone to severe allergies. Several formulations have been modified, including paclitaxel liposome and albumin paclitaxel.^15^ The first nano‐platform commercialized as a drug delivery system was liposomal formulation. Liposomes can modulate paclitaxel toxicity without affecting antitumor activity compared to cremophor‐based paclitaxel (PTX) at the same dosage; it has the advantage of not requiring a high dose of hormone pre‐treatment by reducing the risk of severe allergic reactions. In addition, it has the characteristics of nano drugs, which are made by encapsulating paclitaxel in liposomes and can directly target the tumor and lymph nodes, with lower heart and bone marrow toxicity.^16^ Currently, paclitaxel liposome has been indicated as a first‐line treatment drug for ovarian cancer, breast cancer, and non‐small cell lung cancer in China. However, there are data regarding ESCC, even fewer data in combination with radiotherapy.^17^, ^18^, ^19^

Therefore, we conducted this retrospective study at our center to gain insights into the relative efficacy and toxicities of paclitaxel liposome combined with radiotherapy in patients with locally advanced ESCC.

## 2 PATIENTS AND METHODS

### 2.1 Patient selection

The clinical data of patients with locally advanced ESCC who received radiotherapy at the Fudan University Shanghai Cancer Center between June 2016 and April 2019 were retrospectively analyzed. We retrospectively reviewed the medical records, RT treatment plans, and diagnostic images of the patients who satisfied the following criteria: (1) 18–75 years of age; (2) histologically verified squamous cell carcinoma; (3) clinical stage III or partial IV (supraclavicular lymph node metastasis, according to the TNM classification of the UICC International Union Against Cancer, 8th edition); (4) Eastern Cooperative Oncology Group performance status (ECOG PS) of 0–2; (5) treatment with concurrent or sequential chemotherapy with TP regimen (paclitaxel liposome plus cisplatin). We excluded patients with: (1) esophageal carcinoma who underwent surgery; (2) incomplete information and follow‐up failure; (3) other active cancers. Another historical control group of inoperable ESCC patients treated with a standard PF regimen (5‐FU plus cisplatin) was illustrated for comparison.^11^ In this cohort, 88 patients in stages II–IVB in the same center between April 2010 and July 2016 were enrolled.

This retrospective study was approved by the institutional review board of the Fudan University Shanghai Cancer Center. Informed consent was obtained from all patients before their initial treatment and was not required for the publication of this study due to the retrospective nature of this study.

### 2.2 Treatment schedule

Radiotherapy protocol: Primary esophageal gross tumor volume (GTV) was defined using endoscopy, barium esophagography, and computed tomography (CT) or positron emission computed tomography. The planning target volume (PTV) was defined as the GTV plus 3 cm of the proximal and distal normal esophagus with a 1.2 cm lateral margin. The intensity‐modulated radiotherapy (IMRT) technique was used. The Pinnacle treatment planning system (Philips Medical System, Pinnacle) was used to design the treatment plan. Irradiation was delivered at 6‐MV photon energy. For the radiation prescription, 60 Gy was administered to the PTV by conventional fractionation in 30 fractions for 6 weeks. Plan optimization was based on a dose–volume histogram (DVH). The prescribed isodose curve covered 95% of the PTV, and the 95% isodose curve covered 99% of the PTV. The maximum dose within the PTV did not exceed 110% of the prescribed dose. The dose constraints allowed a maximum dose of <45 Gy to the spinal cord, and the lung volume received 20 Gy, not exceeding 30%. A mean dose of <30 Gy was administered to the heart. Tissue inhomogeneity corrections were applied to all the dose calculations. Cone beam CT was performed weekly to verify the tumor position.

Chemotherapy protocol: The TP chemotherapy regimen used was paclitaxel liposome 135 mg/m^2^ d1 plus cisplatin 25 mg/m^2^ d1‐3 every 3 weeks. The PF group was 5‐FU 600 mg/m^2^ d1‐3 and cisplatin 25 mg/m^2^ d1‐3 every 4 weeks. Chemotherapy was simultaneously supplemented with antiemetic, anti‐allergy, and other symptomatic treatments to reduce side effects. In the TP group, induction chemotherapy was defined as one cycle of TP regimen chemotherapy before radiotherapy; concurrent chemotherapy was defined as two cycles of TP regimen chemotherapy concurrent with radiotherapy; consolidation chemotherapy was defined as one cycle of chemotherapy after radiotherapy. The cycles of individualized chemotherapy were adjusted based on patients' tolerance to chemotherapy.

### 2.3 Response evaluation

The method for evaluating the treatment efficacy of esophageal cancer is based on the response evaluation criteria in solid tumors (RECIST 1.1),^20^ which is based on gastroscopy and CT. However, a gastroscopy was replaced by esophagography at our center. We classified the responses of patients with esophagography after radiotherapy into three grades: complete response (CR), partial response (PR), and no response (NR), by the degree of esophageal stenosis, the smoothness of the esophageal wall, and the recovery of the esophageal mucosa. The definition of CR by esophagogram was as follows: the esophagogram showed smooth esophageal edges, no obvious esophageal stenosis (the width of lesion stenosis was larger than 2/3 of the adjacent esophagus), and the mucosal phase returned to normal, only allowing a slight mucosal phase disorder. The definition of PR by esophagogram was as follows: The filling phase shows that the esophageal stenosis was significantly relieved, but the width of the lesion stenosis was less than 2/3 of the adjacent esophagus; the esophageal wall was slightly unsmooth, and the mucosal phase showed that the mucosal fold was restored but was still obviously disordered. NR was defined as lesions that were significantly residual or aggravated.

By combining CT and esophagogram, the assessment standard of CR: esophagogram showed CR; CT images showed the maximum thickness of esophageal primary lesion ≤1.2 cm and the shortest diameter of metastatic lymph node ≤1.0 cm. The criteria for judging PR by esophagography and CT were as follows: (1) esophagogram indicates CR, while CT images show that the thickness of the esophageal wall was greater than 1.2 cm or the shortest diameter of lymph nodes was larger than 1 cm; or (2) esophagogram indicated PR. Criteria for determining NR by esophagography and CT: NR assessed by esophagography; the original lesion increased by more than 20% as evaluated by CT, or new lesions appeared. The evaluation schematic diagram for response by esophagogram is shown in the (Figures S1–S5).

### 2.4 Statistical analysis

Adverse events after treatment were evaluated according to the Common Terminology Criteria for Adverse Events (CTCAE 5.0) after treatment.^21^ Overall survival (OS) was defined as the time between the first treatment session and death. Progression‐free survival (PFS) is the time between the first treatment session and progression or death. Local relapse‐free survival (LRFS) was defined as the time between the first treatment session and local or regional relapse. Distant metastasis‐free survival (DMFS) was defined as the time between the first treatment session and metastasis. Survival was estimated using the Kaplan–Meier method and compared between different clinical factors in univariate and multivariate analyses using the Cox regression model. Factors with a *p‐*value <0.05 in the univariate analyses were included in the multivariate analysis. Response evaluation was estimated by the logistic regression analysis method. Fisher's exact tests were used to evaluate the differences in toxicity between the TP and FP groups.

All statistical analyses were conducted using IBM SPSS Statistics for Windows version 22.0 (IBM Corp.), and statistical significance was indicated by two‐sided *p‐*values with α set at 0.05.

## 3 RESULTS

### 3.1 Patient baseline and clinical characteristics

Thirty‐nine patients with 39 locally advanced ESCC who received chemoradiotherapy were eligible for enrollment. The median age of the patients was 65 years (range:17–75). Detailed patient characteristics and treatment details are provided in Table 1. One of the 39 patients did not complete radiotherapy due to severe obstruction and vomiting. Seven patients were treated with sequential radiotherapy and chemotherapy because of the poor intolerance to induction chemotherapy.

**TABLE 1:** Baseline and treatment characteristics (*n* = 39)

Variable	*n* (%)
Age (years)	
Median	65
Range	18–75
Sex	
Male	31 (79.5)
Female	8 (20.5)
ECOG performance status	
0	1 (2.6)
1	37 (94.9)
2	1 (2.6)
Main tumor location	
Cervical	2 (5.1)
Upper thoracic	12 (30.8)
Middle thoracic	11 (28.2)
Lower thoracic	14 (35.9)
TNM stage (UICC 8th)	
IIIB	18 (46.2)
IVA	20 (51.3)
IVB	1 (2.6)
Tumor length	
<7 cm	30 (76.9)
≥7 cm	9 (23.1)
Eating condition	
Normal	3 (7.7)
Semi‐liquid diet	18 (46.2)
Liquid diet	17 (43.6)
Dysphagia	1 (2.6)
Loss of weight	
<5%	27 (69.2)
5–10%	7 (17.9)
>10%	5 (12.8)
Radiation dose (Gy)	
Median	60
Range	48–66
Radiation Time (days)	
Median	44
Range	36–106
Interruption of radiotherapy	
Yes	1 (2.6)
No	38 (97.4)
Total chemotherapy cycle	
<4	17 (43.6)
4	17 (43.6)
>4	5 (12.8)
Induction chemotherapy cycle	
0	3 (7.7)
1	21 (53.8)
2	10 (25.6)
3	5 (12.8)
Concurrent chemotherapy cycle	
0	7 (17.9)
1	16 (41.0)
2	16 (41.0)
Consolidation chemotherapy cycle	
0	21 (53.8)
1	10 (25.6)
2	5 (12.8)
3	2 (5.1)
4	1 (2.6)
Chemotherapy reduction	
Yes	7 (17.9)
No	32 (82.1)

Abbreviations: ECOG, Eastern Cooperative Oncology Group; UICC, International Union Against Cancer.

### 3.2 Survival

**FIGURE 1:**
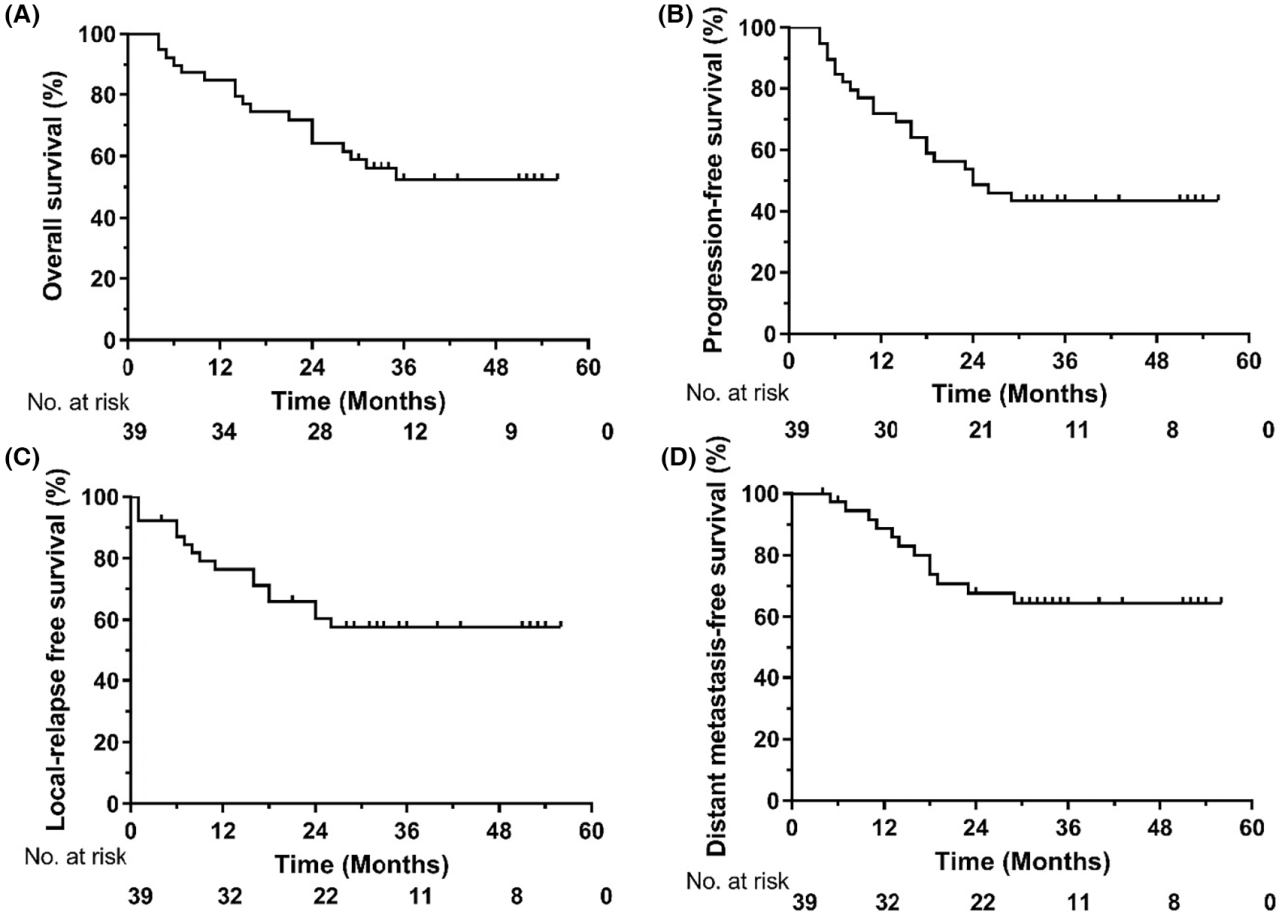
The survival rates of all patients. (A) Overall survival, (B) progression‐free survival, (C) local relapse‐free survival, and (D) distant metastasis‐free survival.

The median follow‐up time was 31.5 months, with a deadline of August 2021 for the final follow‐up. At the time of our analyses, 27 patients were still alive, and 19 patients had no evidence of disease progression. Kaplan–Meier curves for OS, PFS, LRFS, and DMFS are shown in Figure 1. The 1‐, 2‐, and 3‐year OS rates were 84.6%, 64.1%, and 56.2%, respectively. The 1‐, 2‐, and 3‐year PFS rates were 71.8%, 43.6%, and 43.6%, respectively. The 1‐, 2‐, and 3‐year LRFS rates were 76.5%, 60.4%, and 57.7%, respectively. The 1‐, 2‐, and 3‐year DMFS rates were 80.0%, 67.7%, and 64.5%, respectively. The median OS time was 38.3 (95% confidence interval [CI]:32.1–45.1) months, and the median PFS time was 32.1 (95% CI:25.4–39.0) months. In the univariate and multiple analyses, age and TNM stage were significant factors for OS (Table 2). We also compared the OS, PFS, LRFS, and DMFS between the TP and PF groups. There were no significant differences in OS, LRFS, PFS, and DMFS between the PF regimen cohort and TP regimen cohort in our center. However, the OS and LRFS rates suggested that the TP regimen was slightly better than the traditional PF regimen, but the results did not reach a statistical difference. The corresponding survival curves are shown in Figure S6.

**TABLE 2:** Factors related to overall survival after treatment

	Univariate analysis		Multivariate analysis	
	HR (95% CI)	*p*‐value	HR (95% CI)	*p*‐value
Sex		0.074		
Male	1			
Female	0.192 (0.024–1.452)			
Age		0.043*		0.047*
≥60 years	1		1	
<60 years	0.155 (0.021–1.164)		0.128 (0.017–0.975)	
Loss of weight		0.996		
<5%	1			
5%–10%	0.942 (0.210–4.215)			
>10%	0.968 (0.162–5.804)			
TNM stage		0.043*		0.021*
III	1		1	
IV	2.819 (0.990–8.027)		3.457 (1.202–9.949)	
Main tumor location		0.521		
Cervical	1			
Upper thoracic	‐			
Middle thoracic	1.437 (0.483–4.281)			
Lower thoracic	0.799 (0.0225–2.836)			
Tumor length		0.272		
≥7 cm	1			
<7 cm	0.561 (0.197–1.596)			
Radiotherapy interruption		0.670		
No	1			
Yes	1.290 (0.399–4.167)			
Total number of chemotherapy cycle		0.295		
≥4	1			
<4	2.808(0.372–21.190)			
Concurrent chemotherapy		0.393		
No	1			
Yes	0.615 (0.199–1.896)			
Consolidation chemotherapy		0.661		
No	1			
Yes	0.806 (0.307–2.118)			
Chemotherapy reduction		0.198		
No	1			
Yes	2.062 (0.669–6.355)			

* In the univariate and multiple analyses, age and TNM stage were significant factors for OS, *p*‐value < 0.05.

### 3.3 Efficacy and patterns of failure

**FIGURE 2:**
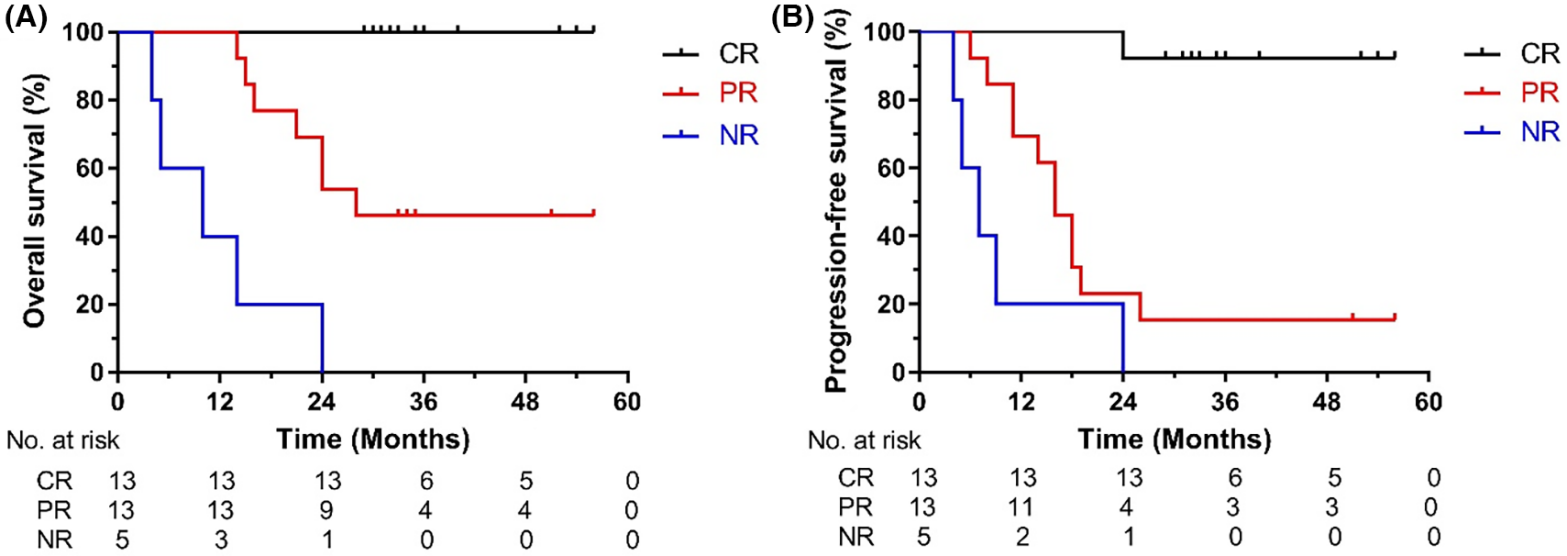
The survival rates for different responses after treatment. (A) Overall survival, (B) progression‐free survival. CR, complete response; NR, no response; PR, partial response.

After excluding eight unevaluable patients, the number of CR, PR, and NR patients was 13 (41.9%), 13 (41.9%), and five (16.1%), respectively. Univariate analysis shows that sex (*p* = 0.064), stage (*p* = 0.086), and concurrent chemotherapy (*p* = 0.064) tended to differ in response, but did not reach statistical significance. The logistic regression analysis results are shown in the Table S1. The 3‐year OS rates for CR, PR, and NR were 100%, 46.2%, and 0%, respectively (*p* = 0.022). The 3‐year PFS rates for CR, PR, and NR were 92.3%, 15.4%, and 0%, respectively (*p* = 0.001). The OS and PFS curves of the three groups are shown in Figure 2.

Simultaneously, we analyzed the failure patterns, as shown in Table 3. At the time of the final analysis, six patients had locoregional progression within the irradiated field, six patients had regional progression outside the irradiation field, and 12 patients had distant metastases, including four in the lungs, four in the bones, two in the liver, one in the pleura, and one in the non‐regional lymph nodes (supraclavicular and hilar lymph nodes).

**TABLE 3:** The failure patterns in 39 ESCC patients after treatment

Failure patterns	*N* (%)
Total patients	39 (100)
Locoregional Pattern 1	
In‐field	6 (15.4)
Out‐field	6 (15.4)
In‐field and out‐field	2 (5.1)
None	25 (64.1)
Locoregional Pattern 2	
Primary tumor region	8 (20.5)
Regional lymph node	6 (15.4)
None	25 (64.1)
Distant metastasis	
Distant organ	11 (28.2)
Distant node	1 (2.6)
None	27 (69.2)

### 3.4 Toxicity

The toxicities of the treatments within 6 months are shown in Table 4. The most frequent acute adverse events for Grades 3 and 4 were leukocytopenia, neutropenia, thrombocytopenia, and esophagitis. Grades 3 and 4 leukocytopenia were observed in nine (23.1%) and seven (17.9%) patients, respectively. Grades 3 and 4 thrombocytopenia, anemia, esophagitis, and pneumonitis occurred in three (7.7%), six (15.4%), four (10.3%), and zero (0%) patients, respectively. We compared the Grades 3 and 4 toxicities between the TP and PF groups (Table S2). The Grades 3 and 4 toxicities of leukocytopenia (41%) and neutropenia (35.9%) in the TP group were higher than in the comparison group, in which the leukocytopenia and neutropenia were 14.8% (*p* = 0.002) and 12.5% (*p* = 0.004), respectively. While the toxicities of anorexia and vomiting in the TP group were lower than in the comparison group, and the differences were statistically significant.

**TABLE 4:** Treatment‐related toxicity within 6 months

Grade	1	2	3	4
*n* = 39	*n* (%)	*n* (%)	*n* (%)	*n* (%)
Hematological				
Leukocytopenia	3 (7.7)	12 (30.8)	9 (23.1)	7 (17.9)
Neutropenia	3 (7.7)	10 (25.6)	2 (5.1)	12 (30.8)
Anemia	17 (43.6)	15 (38.5)	6 (15.4)	0 (0)
Thrombocytopenia	15 (38.5)	5 (12.8)	2 (5.1)	1 (2.6)
Lymphocytopenia	2 (5.1)	6 (15.4)	23 (59.0)	8 (20.5)
Transaminase increased	10 (25.6)	1 (2.6)	0 (0)	0 (0)
Creatinine increased	0 (0)	0 (0)	0 (0)	0 (0)
Non‐hematological				
Anorexia	6 (15.4)	0 (0)	0 (0)	0 (0)
Vomit	23 (59.0)	4 (10.3)	0 (0)	0 (0)
Esophagitis	5 (12.8)	23 (59.0)	1 (2.6)	3 (7.7)
Acute pneumonia	11 (28.2)	1 (2.6)	0 (0)	0 (0)

## 4 DISCUSSION

Paclitaxel is not an indication drug for esophageal cancer, but it was commonly used as a chemotherapy agent in clinical practice and in recent clinical trials.^10^, ^12^ In principle, clinical trials should be conducted as prospective studies for drugs that do not have an indication. However, off‐label drug application is quite common in clinical practice, especially in cases with a relative lack of indicated drugs. Currently, esophageal cancer treatments are in the era of immunotherapy, and few medical companies are interested in spending huge amounts of money to broaden the indications of classical chemotherapy drugs. The price of paclitaxel liposome in China has been reduced and is close to the ordinary paclitaxel. Paclitaxel liposome's application in esophageal cancer has been more common, but the data on its efficacy and safety are still relatively scarce. Therefore, we conducted this retrospective study at our center to gain insights into the relative efficacy and toxicities of paclitaxel liposome combined with radiotherapy in patients with locally advanced ESCC.

In this retrospective study, using chemoradiotherapy with paclitaxel liposome plus cisplatin in 39 locally advanced ESCC patients, the clinical CR rate was 41.9%, and the 3‐year PFS and OS were 43.6% and 56.2%, respectively; indicating good clinical outcomes. There were no significant differences in OS, LRFS, PFS, and DMFS between the PF regimen cohort and TP regimen cohort in our center. However, the OS and LRFS rates suggested that the TP regimen was slightly better than the traditional PF regimen, but the results did not reach a statistical difference. Recently, three phase III randomized controlled studies from China showed that the 3‐year survival rate of ESCC patients was up to 50% in the era of intensity‐modulated radiotherapy.^10^, ^12^, ^22^ However, during the same period, the 3‐year survival rate of concurrent chemoradiotherapy (CRT) in Western countries was approximately 30%–40%.^8^, ^9^, ^23^ In addition, clinical studies from China showed that the PFS curve entered a plateau after 2 years of treatment, and the OS curve entered a plateau after 3 years of treatment. In contrast, the survival curve was continuously downward after treatment in Western countries.^7^, ^8^, ^9^, ^23^ Because the patient characteristics and radiation technology may differ in different countries and clinical trials, an international multicenter randomized controlled clinical trial should be conducted to verify these differences.

The survival results in our study were similar to those of two other studies on paclitaxel liposome combined with platinum‐based chemoradiotherapy.^24^, ^25^ Paclitaxel liposome did not appear to significantly improve efficacy compared to conventional paclitaxel. Recently, a phase III clinical study reported a 3‐year survival rate of 60% using paclitaxel combined with cisplatin chemoradiation at our center.^12^ Nevertheless, in a phase II study by the same research group, the 3‐year survival rate of the TP regimen was only 40%. This may be because some patients were treated with three‐dimensional conformal radiotherapy (3DCRT) in a previous study.^26^ In another clinical study using a TP regimen combined with 3DCRT, the 3‐year survival rate was only 28%.^8^ Advances in radiotherapy technology may be the main reason for these discrepancies. A retrospective study demonstrated that IMRT could improve local control and survival rates compared to 3DCRT.^27^

Herein, the safety of the treatment was satisfactory. The most common Grade IV adverse event was neutropenia (30%), which was lower than that of cremophor‐based paclitaxel (43.0%).^12^ There was no Grade III/IV radiation pneumonia, and only one case of Grade II radiation pneumonia occurred. Compared with the PF regimen, the Grade 3 and 4 toxicities of TP were mainly leukopenia and neutropenia, and the TP regimen had a lower rate of gastrointestinal discomfort. There was no difference in other toxicities. In previous studies, synchronous paclitaxel administration increased the risk of radiation pneumonia.^10^, ^28^ We hypothesized that this was related to the frequency of paclitaxel administration. Among the three paclitaxel‐based combination regimens, symptomatic radiation pneumonia was significantly increased in TC and TF on weekly regimens compared with TP on three‐week regimens (21.5%, 26.1%, and 4.7%, respectively; *p* < 0.001).^12^ Due to the limitations of this retrospective study, the adverse reactions reported in this study were not comprehensively included, such as peripheral neuropathy. In other studies, liposome autophagy significantly reduced allergy, skin rash, gastrointestinal toxicity, bone marrow suppression, muscle/joint pain, and peripheral neuropathy compared with conventional paclitaxel.^29^, ^30^

In this study, we assessed the efficacy evaluation methods of esophagography combined with CT, which can effectively determine patients' prognoses. The 3‐year PFS rate of patients who achieved CR was 92.3%, whereas the PFS rate of patients who did not achieve CR was only 15.3%. This significant result lays the foundation for future individualized treatments. This CR standard can also be used as the primary endpoint of phase II studies to improve the efficiency of clinical studies. At present, the criteria for early evaluation of efficacy for esophageal cancer are RESCIST 1.1^20^ using gastroscopy and CT. The standard of CR is as follows: the thickness of the esophageal wall is less than 1 cm, the shortest diameter of all lymph nodes is less than 1 cm, no new metastases are observed, and the lesion disappears under gastroscopy. However, this standard cannot adequately distinguish the prognosis of patients,^31^ partly because the CR standard of esophageal thickness less than 1 cm is too strict. Esophageal cancer radiotherapy is often accompanied by edema; as a result, the esophageal thickness will not be reduced to normal in a short time. Recently, MRI has been used to evaluate the efficacy of radiotherapy and chemotherapy for esophageal cancer.^32^, ^33^, ^34^ The recovery of the esophageal structure in the T2 phase with an ADC value ≥2.64 × 10^−3^ mm^2^/s became the new CR standard, which no longer limits the thickness of the esophageal wall. The new CR standard based on MRI can effectively distinguish the prognosis of patients.^31^ However, the shortcomings of MRI are that the ADC values measured by different parameters at different centers are not replicable, and the cost is high. The application of esophagograms to evaluate early curative effects has a long history in China and has accumulated a lot of evidence. An esophagogram can show whether the outline of the esophagus is narrow and whether the esophageal mucosal folds are restored. Combining CT and esophagogram findings, such as a smooth esophageal wall, no significant stenosis, and recovery of mucosal folds, the early prediction of long‐term prognosis was successfully achieved. Unfortunately, the sample size in the present study was small and requires verification with a larger sample size in the future.

The TPF triplet regimen (docetaxel, cisplatin, and 5‐FU) has a trend of further improving treatment efficacy compared with the traditional doublet regimen.^35^, ^36^ In a phase II study, concurrent chemoradiotherapy with TPF achieved a clinical CR rate of 52.4%, much higher than the previously reported CR rate of 20%.^9^ However, the high hematological toxicity of the TPF triplet regimen limits its clinical application. Paclitaxel liposome can significantly reduce bone marrow toxicity compared to docetaxel and is expected to replace docetaxel in the triplet regimen. In a nasopharyngeal cancer study, the incidence of Grade III‐IV neutropenia in paclitaxel liposome‐based and docetaxel‐based TPF regimens was 21.4% and 47.0%, respectively (*p* < 0.001), with no difference in efficacy.^37^ Esophageal cancer treatment has entered the era of immunotherapy. There are currently three randomized controlled phase III studies exploring the efficacy and toxicity of combining PD‐1 immune checkpoint inhibitors with concurrent chemoradiotherapy. In one published phase Ib study, concurrent chemoradiotherapy plus synchronous and maintenance treatment with camrelizumab achieved a 2‐year PFS rate of 65%, which was the highest in published data.^38^ Our research group has also conducted a phase II clinical study to explore the feasibility of TPF combined with PD‐1 immune checkpoint inhibitors in patients with ESCC.

This study had some limitations. First, the small sample size may bias the results. To obtain high‐level clinical evidence, prospective randomized, controlled, multi‐center clinical studies should be conducted to further confirm current study findings and conclusions. Second, non‐hematological toxicities such as neurotoxicity are subjective and cannot be accurately assessed; therefore, they were not included in our toxicity analysis. In future studies, attention will be paid to the collection of toxicity data.

## 5 CONCLUSIONS

In this retrospective study, we analyzed the feasibility and efficacy of paclitaxel‐liposome‐based chemoradiotherapy in 39 patients with locally advanced ESCC. The clinical CR rate was 41.9%, and the 3‐year PFS and OS rates were 43.6% and 56.2%, respectively. The Grades III–IV neutropenia rate was 35.9%, and only one case of Grade II radiation pneumonia occurred. Our study suggests that paclitaxel liposome is safe and effective for treating locally advanced ESCC.

## AUTHOR CONTRIBUTIONS

**Qiong Yi:** Data curation (equal); formal analysis (equal); writing – original draft (equal). **Canyu Liu:** Data curation (equal); formal analysis (equal); software (equal); validation (equal); writing – original draft (equal). **Yingshan Cui:** Formal analysis (supporting); investigation (equal); software (supporting). **Yangguang Yang:** Formal analysis (supporting); validation (supporting). **Yaqi Li:** Investigation (supporting); writing – original draft (supporting). **Xingwen Fan:** Conceptualization (lead); investigation (lead); methodology (lead); project administration (lead); validation (equal); writing – review and editing (lead).

## FUNDING INFORMATION

This work was supported by the Key Clinical Specialty Project of Shanghai.

## CONFLICT OF INTEREST

All authors declare no conflicts of interest.

## ETHICAL APPROVAL AND INFORMED CONSENT

All procedures performed in this study involving human participants were in accordance with the ethical standards of the Fudan University Shanghai Cancer Center Ethics Committee and with the 1964 Helsinki Declaration and its later amendments or comparable ethical standards.

## Supporting information


Data S1

## Data Availability

The datasets used and/or analyzed during the current study are available from the corresponding author upon reasonable request.
