# Supplementary material for: Chemoradiotherapy with paclitaxel liposome plus cisplatin for locally advanced esophageal squamous cell carcinoma: A retrospective analysis
Source: Cancer Med. 2022 Nov 22;12(6):6477–87. doi: 10.1002/cam4.5416 (PMC10067117; doi:10.1002/cam4.5416)
Supplement: Supplementary file 1 — Data S1 [file CAM4-12-6477-s001.docx]

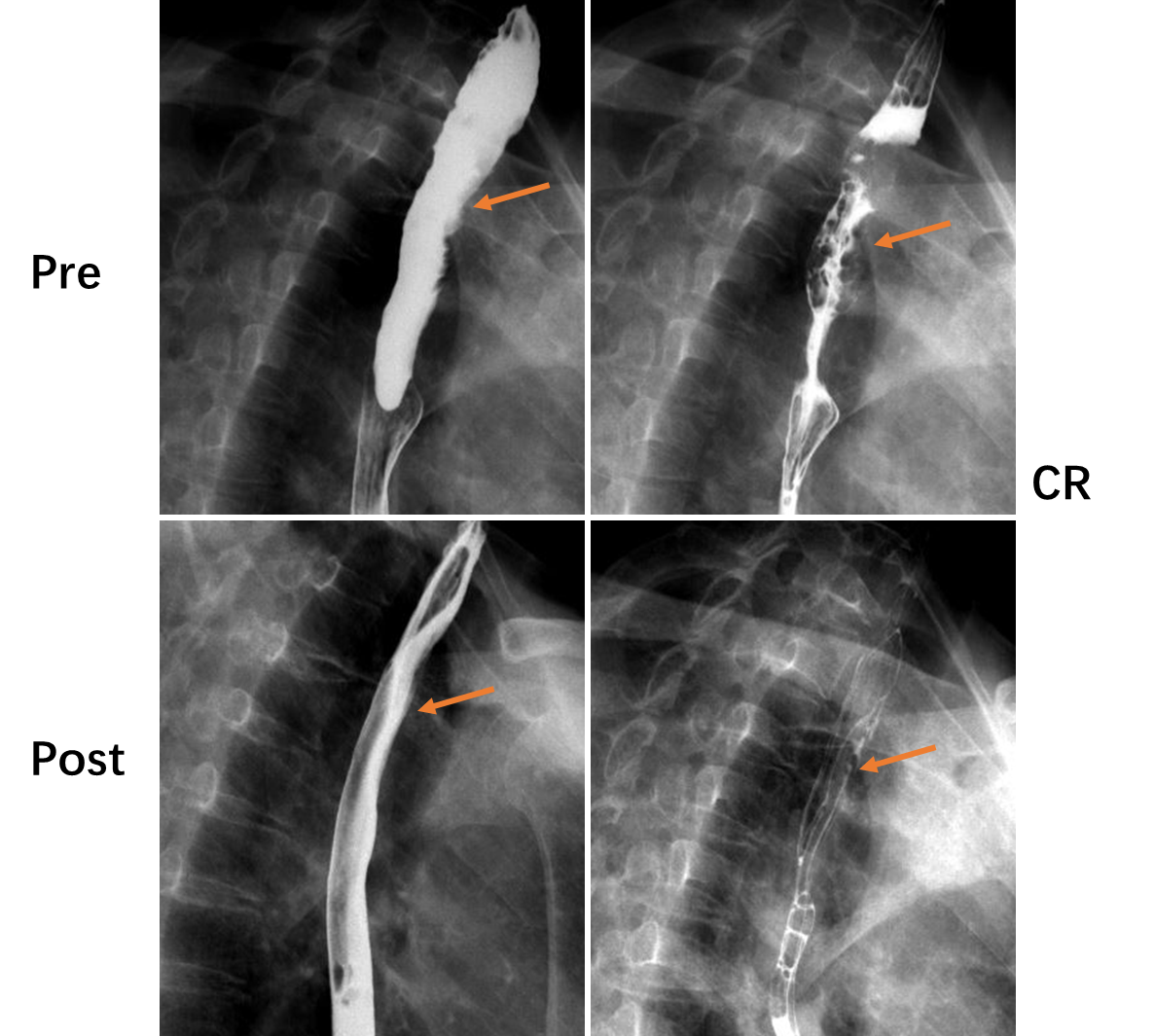


Figure S1. Classic picture of a complete response by esophagography.


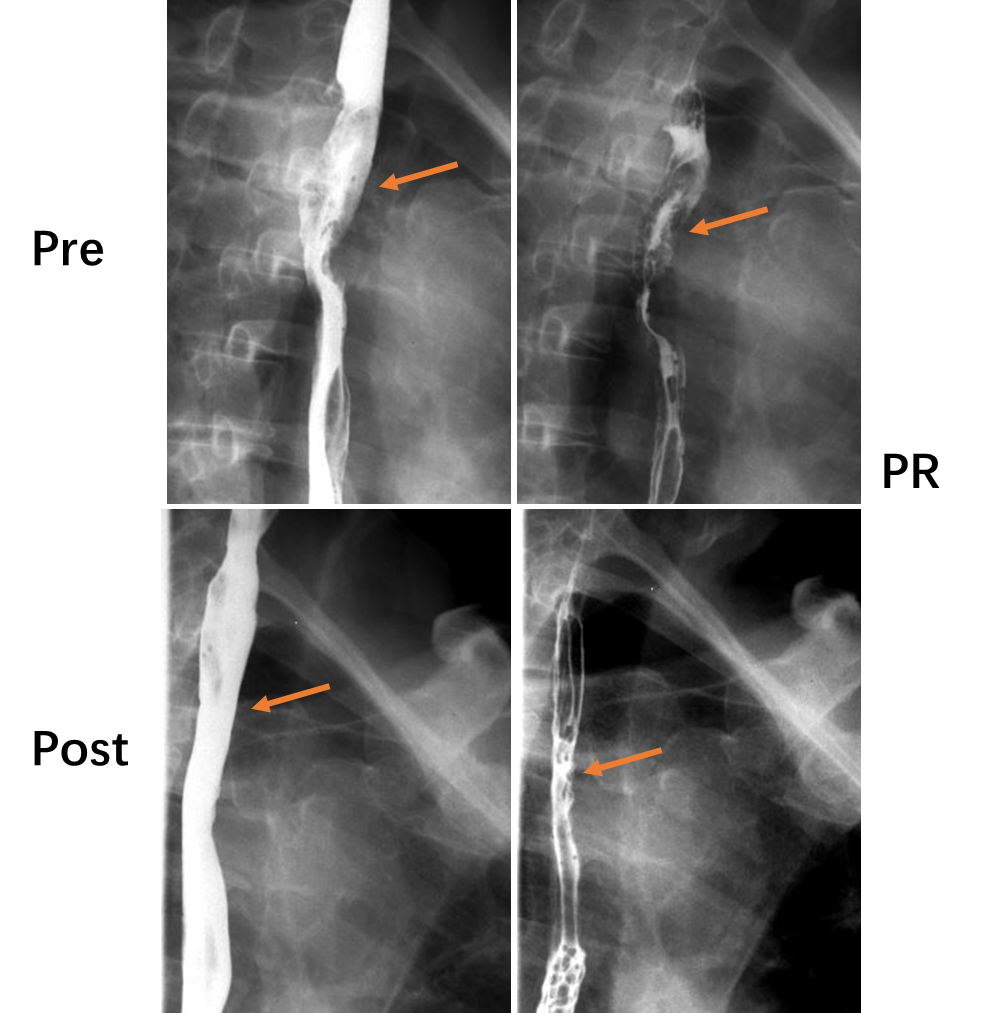


Figure S2. Classic picture of a partial response by esophagography because a mucosal fold is still an obvious disorder.


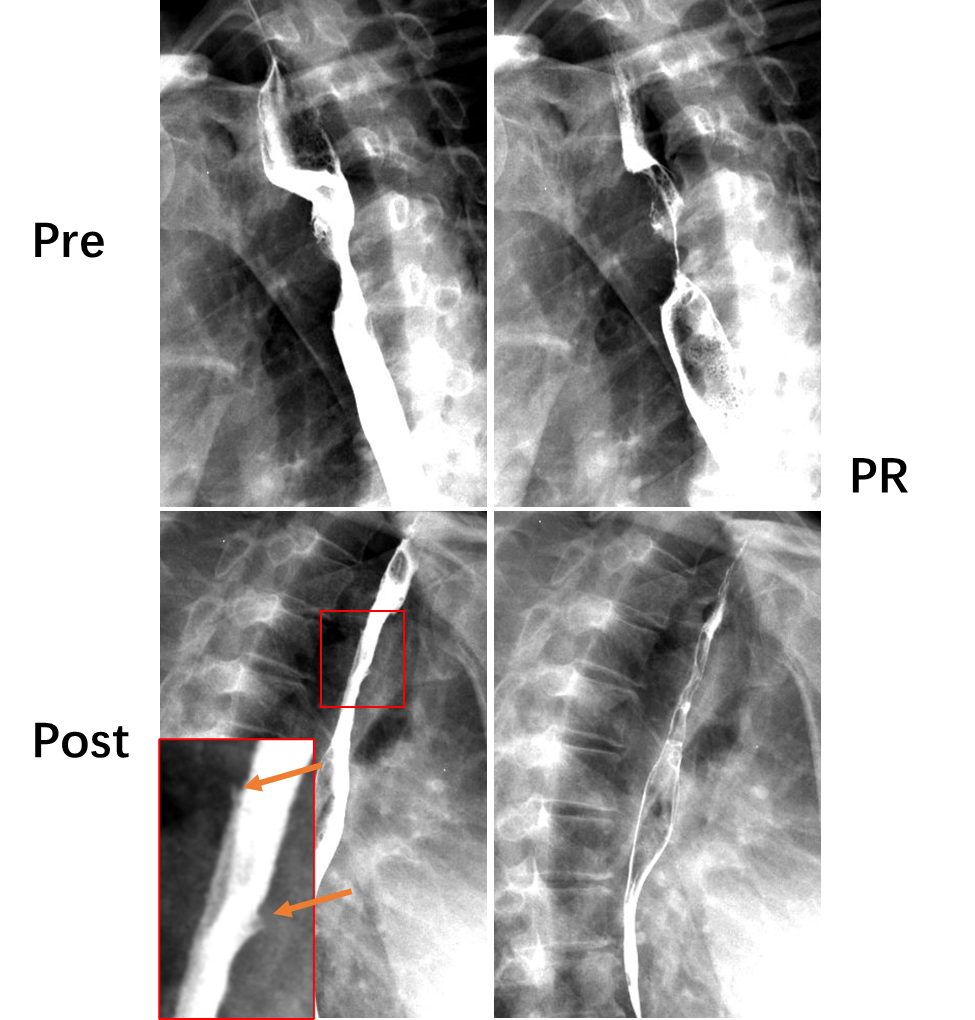


Figure S3. Classic picture of a partial response by esophagography because the esophageal wall is a little unsmooth.


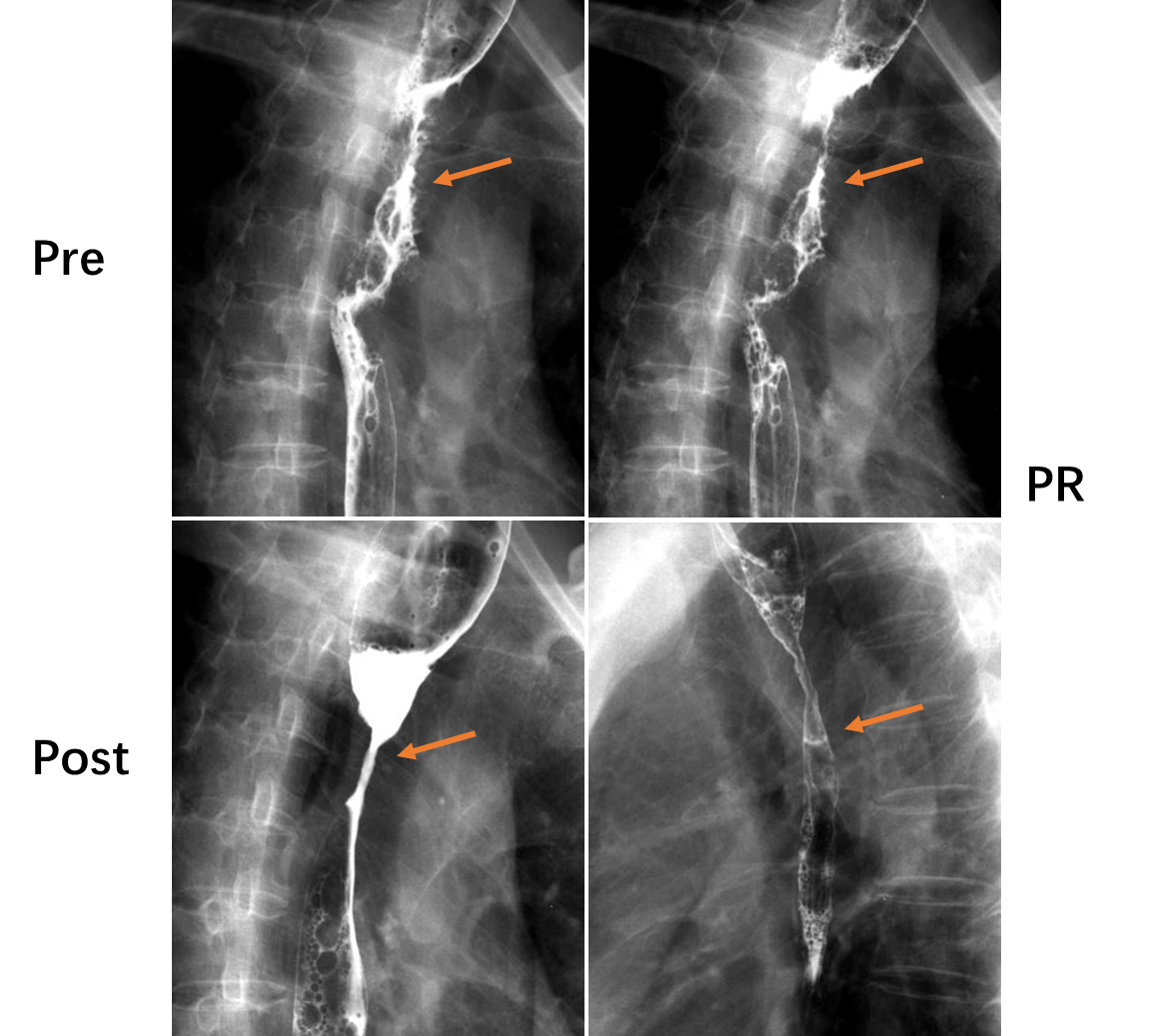
Figure S4. Classic picture of a partial response by esophagography because the width of the lesion stenosis is less than 2/3 of the adjacent esophagus.


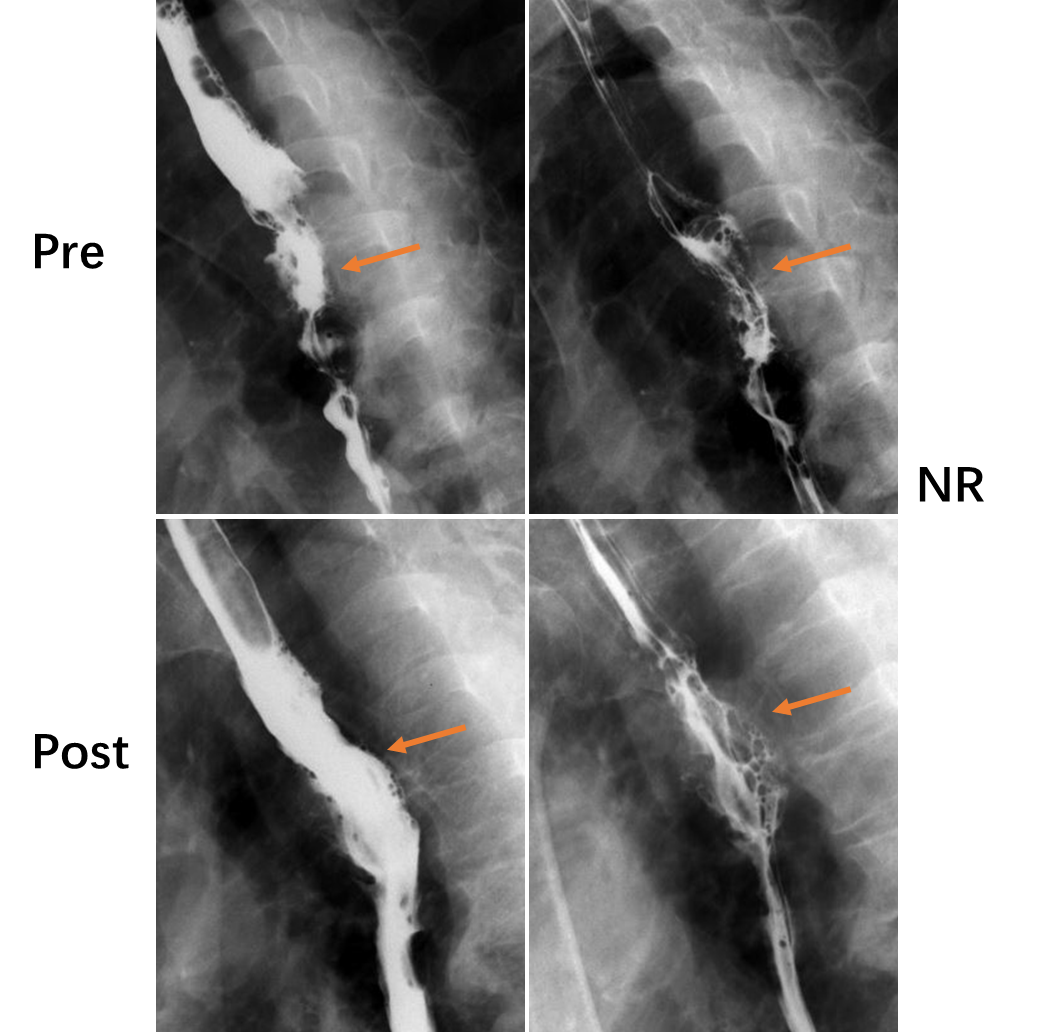


Figure S5. Classic picture of no response by esophagography.


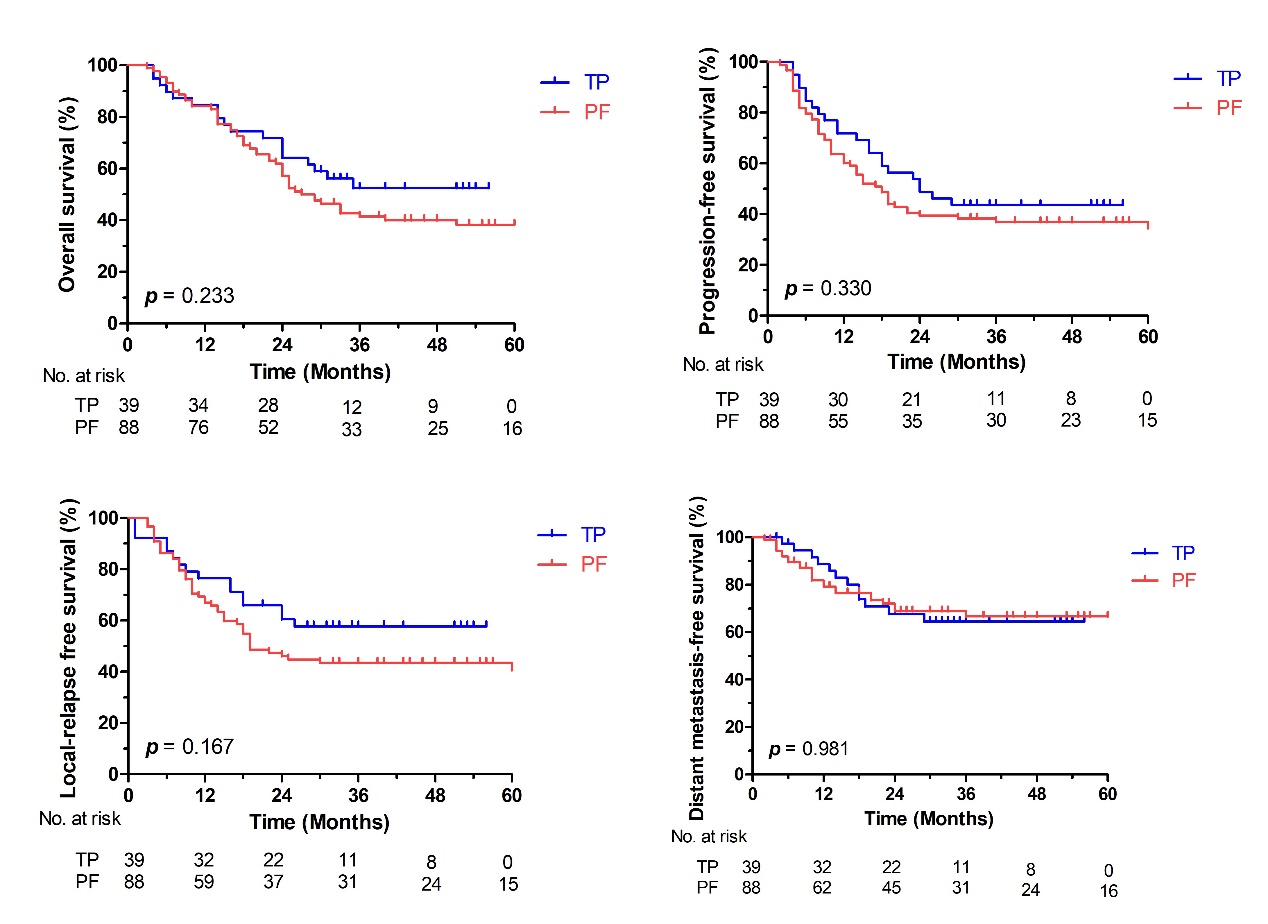


Figure S6. The survival rates of all patients in the TP and PF groups. The blue curves represent the TP group, and the red curves represent the PF group. A) overall survival, B) progression-free survival, C) local relapse-free survival, and D) distant metastasis-free survival.

Table S1. Clinical factors related to response (CR and non-CR)

| **Logistic analysis** | | | | | | | | | |
| --- | --- | --- | --- | --- | --- | --- | --- | --- | --- |
|  | | B | S.E. | Wald | Df | P value | Exp(B) | 95% EXP(B) | |
|  |  |  |  |  |  |  |  | Lower limit | Upper limit |
|  | Sex | 10.741 | 5.809 | 3.419 | 1 | 0.064 | 46209.648 | 0.525 | 4063861385.092 |
|  | Age | 4.756 | 3.740 | 1.617 | 1 | 0.203 | 116.287 | 0.076 | 177354.569 |
|  | Loss of weight | 2.483 | 1.701 | 2.129 | 1 | 0.145 | 11.973 | 0.427 | 336.050 |
|  | TNM stage | -3.964 | 2.309 | 2.947 | 1 | 0.086 | 0.019 | 0.000 | 1.754 |
|  | Tumor length | 1.741 | 2.064 | 0.711 | 1 | 0.399 | 5.701 | 0.100 | 325.982 |
|  | Main location | 1.023 | 0.841 | 1.480 | 1 | 0.224 | 2.781 | 0.535 | 14.441 |
|  | Total number of chemotherapy cycle | -0.211 | 2.757 | 0.006 | 1 | 0.939 | 0.810 | 0.004 | 180.144 |
|  | Concurrent chemotherapy | 7.017 | 3.782 | 3.442 | 1 | 0.064 | 1114.889 | 0.673 | 1847252.505 |
|  | Consolidation chemotherapy | -3.085 | 3.072 | 1.009 | 1 | 0.315 | 0.046 | 0.000 | 18.835 |
|  | Chemotherapy reduction | 6.530 | 4.208 | 2.409 | 1 | 0.121 | 685.437 | 0.180 | 2615143.081 |

Table S2. Treatment-related toxicity in the TP and PF groups

|  | **TP** | **PF** | ***p*-value** |
| --- | --- | --- | --- |
| Hematological (Grade3/4) |  |  |  |
| Leukocytopenia | 16 (41.0) | 13 (14.8) | 0.002* |
| Neutropenia | 14 (35.9) | 11 (12.5) | 0.004* |
| Anemia | 6 (15.4) | 7 (8.0) | 0.217 |
| Thrombocytopenia | 3 (7.7) | 9 (10.2) | 0.754 |
| Creatinine increased | 0 (0) | 0 (0) | - |
| Non-ematologica l(Grade3/4) |  |  |  |
| Anorexia | 0 (0) | 18 (20.5) | 0.002* |
| Vomit | 0 (0) | 13 (14.8) | 0.009* |
| Esophagitis | 4 (10.3) | 8 (9.0) | 1 |
| Acute pneumonia | 0 (0) | 2 (2.3) | 1 |
